# Mutation detection and minimum inhibitory concentration determination against linezolid and clofazimine in confirmed XDR-TB clinical isolates

**DOI:** 10.1186/s12866-022-02622-x

**Published:** 2022-10-03

**Authors:** Kamal Singh, Swati Sharma, Tuhina Banerjee, Ankush Gupta, Shampa Anupurba

**Affiliations:** 1 Department of Microbiology, Institute of Medical Sciences, Banaras Hindu University, Varanasi, Uttar Pradesh India; 2 Department of Biochemistry, Institute of Science, Banaras Hindu University, Varanasi, Uttar Pradesh India

**Keywords:** GenoTypeMTBDR*sl* v.2.0 assay, REMA method, *rplC* gene, *Rv0678* gene

## Abstract

**Background:**

The emergence of multidrug-resistant tuberculosis (MDR-TB) has complicated the situation due to the decline in potency of second-line anti-tubercular drugs. This limits the treatment option for extensively drug-resistant tuberculosis (XDR-TB). The aim of this study was to determine and compare the minimum inhibitory concentration (MIC) by agar dilution and resazurin microtiter assay (REMA) along with the detection of mutations against linezolid and clofazimine in confirmed XDR-TB clinical isolates.

**Results:**

A total of 169 isolates were found positive for *Mycobacterium tuberculosis* complex (MTBC). The MIC was determined by agar dilution and REMA methods. The isolates which showed non-susceptibility were further subjected to mutation detection by targeting *rplC* gene (linezolid) and *Rv0678* gene (clofazimine). The MIC for linezolid ranged from 0.125 µg/ml to > 2 µg/ml and for clofazimine from 0.25 µg/ml to > 4 µg/ml. The MIC_50_ and MIC_90_ for linezolid were 0.5 µg/ml and 1 µg/ml respectively while for clofazimine both were 1 µg/ml. The essential and categorical agreement for linezolid was 97.63% and 95.26% and for clofazimine, both were 100%. The sequencing result of the *rplC* gene revealed a point mutation at position 460 bp, where thymine (T) was substituted for cytosine (C) while seven mutations were noted between 46 to 220 bp in *Rv0678 gene.*

**Conclusion:**

REMA method has been found to be more suitable in comparison to the agar dilution method due to lesser turnaround time. Mutations in *rplC* and *Rv0678* genes were reasons for drug resistance against linezolid and clofazimine respectively.

**Supplementary Information:**

The online version contains supplementary material available at 10.1186/s12866-022-02622-x.

## Background

The emergence of multidrug-resistant tuberculosis (MDR-TB) has complicated the present global scenario. A steep decline in potency of second-line anti-tubercular drugs against MDR-TB strains has been described in a handful of observational studies [1–3]. Consequently, limited options are left for treating patients with drug-resistant tuberculosis (DR-TB) and particularly extensively drug-resistant TB (XDR-TB). Furthermore, complicating the existing situation is an ever-increasing burden of immuno-compromised population, like the patients living with HIV-AIDS and DR-TB, in whom the mortality is high [4, 5]. The drug susceptibility report of anti-tubercular drugs plays a crucial role for the treatment of disease. The increase of drug resistance in TB requires improved treatment regimens, thereby creating a need for new drugs with different modes of action. Although development of new anti-tubercular drugs is time-consuming and costly, great success has been made in the world's anti-TB drug pipeline [6]. Drugs that are used for the treatment of DR-TB include later generation fluoroquinolones (levofloxacin, moxifloxacin), linezolid, amoxicillin-clavulanate, clarithromycin, thioridazine, clofazimine, bedaquline, and delamanid [7–9]. Traditionally, drug susceptibility testing (DST) for *Mycobacterium tuberculosis complex* (MTBC) has relied on testing a single, critical concentration (CC) that is employed to differentiate resistant from susceptible strains of MTBC and is specific for every anti-TB agent and test method. However, the definitions of CC for MTBC DST have evolved, as have the description of phenotypically wild type (pWT) vs phenotypically non-wild type (pNWT) strains of MTBC [10].

Recent studies have shown that the mycobacterium growth indicator tube (MGIT960); automated liquid medium testing method has become the international gold standard for second-line drug susceptibility testing of MDR and XDR-TB isolates [11–14]. However, all automated processes discriminate between susceptible and resistant and do not determine the minimum inhibitory concentration (MIC) [15].The evaluations of MIC for standard anti-tubercular drugs are important because it will quantify the exact MIC of the clinical isolates. The use of solid media assays (i.e., Lowenstein-Jensen or agar-based 7H10 and 7H11) and liquid media assay (BACTEC MGIT) to determine MICs are relatively time-consuming and costly. In 2002, resazurin microtiter assay (REMA) was developed as a simple, low-cost, highly sensitive and specific method which quickly determined the MICs of first- and second-line anti-TB drugs for *M. tuberculosis* [16, 17]. With this in mind, the study was designed to determine and compare the MIC by agar dilution and REMA method along with the detection of mutations against linezolid and clofazimine in confirmed XDR-TB clinical isolates.

## Results

A total of 169 isolates from 188 sputum specimens were found positive for MTBC. The rest 19 cultures were contaminated. The MIC for linezolid ranged from 0.125 µg/ml to > 2 µg/ml and for clofazimine from 0.25 µg/ml to > 4 µg/ml. The MIC_50_ and MIC_90_ calculated for linezolid was 0.5 µg/ml and 1 µg/ml, respectively. However, the MIC_50_ and MIC_90_ calculated for clofazimine was 1 µg/ml for both 50% and 90% population. The detailed comparative MIC result for linezolid and clofazimine by agar dilution and REMA method has been shown in Tables 1 and 2 and representative plate images showing the MICs have been shown in Supplementary Figs. 1 and 2. The essential and categorical agreement for linezolid was 97.63% and 95.26% respectively with 4.73% minor error. The essential and categorical agreement for clofazimine was 100% with no error as shown in Table 3.

**Table 1:** Comparative MIC result for linezolid by agar dilution and REMA method

	**Gold Standard MIC Method (agar dilution method)**						
**New MIC Method** **(REMA Method)**		**0.125**	**0.25**	**0.5**	**1**	**2**	**>2**
	**0.125**	27	0	0	0	0	0
	**0.25**	0	39	4	0	0	0
	**0.5**	0	0	43	4	0	0
	**1**	4	0	0	46	0	0
	**2**	0	0	0	0	0	0
	**>2**	0	0	0	0	0	2

**Table 2:** Comparative MIC result for clofazimine by agar dilution and REMA method

	**Gold Standard MIC Method (agar dilution method)**						
**New MIC Method** **(REMA Method)**		**0.25**	**0.5**	**1**	**2**	**4**	**>4**
	**0.25**	27	0	0	0	0	0
	**0.5**	1	52	0	0	0	0
	**1**	0	0	87	0	0	0
	**2**	0	0	0	0	0	0
	**4**	0	0	0	0	0	0
	**>4**	0	0	0	0	0	2

**Table 3:** Summary of essential agreement (EA) and categorical agreement (CA) for REMA method compared with MICs by agar dilution method

Test isolates (*n* = 169)	REMA
**Linezolid**	
EA(%)	97.63
CA (%)	95.26
No. of minor error	8 (4.73%)
**Clofazimine**	
EA(%)	100
CA (%)	100

**Fig. 1:**
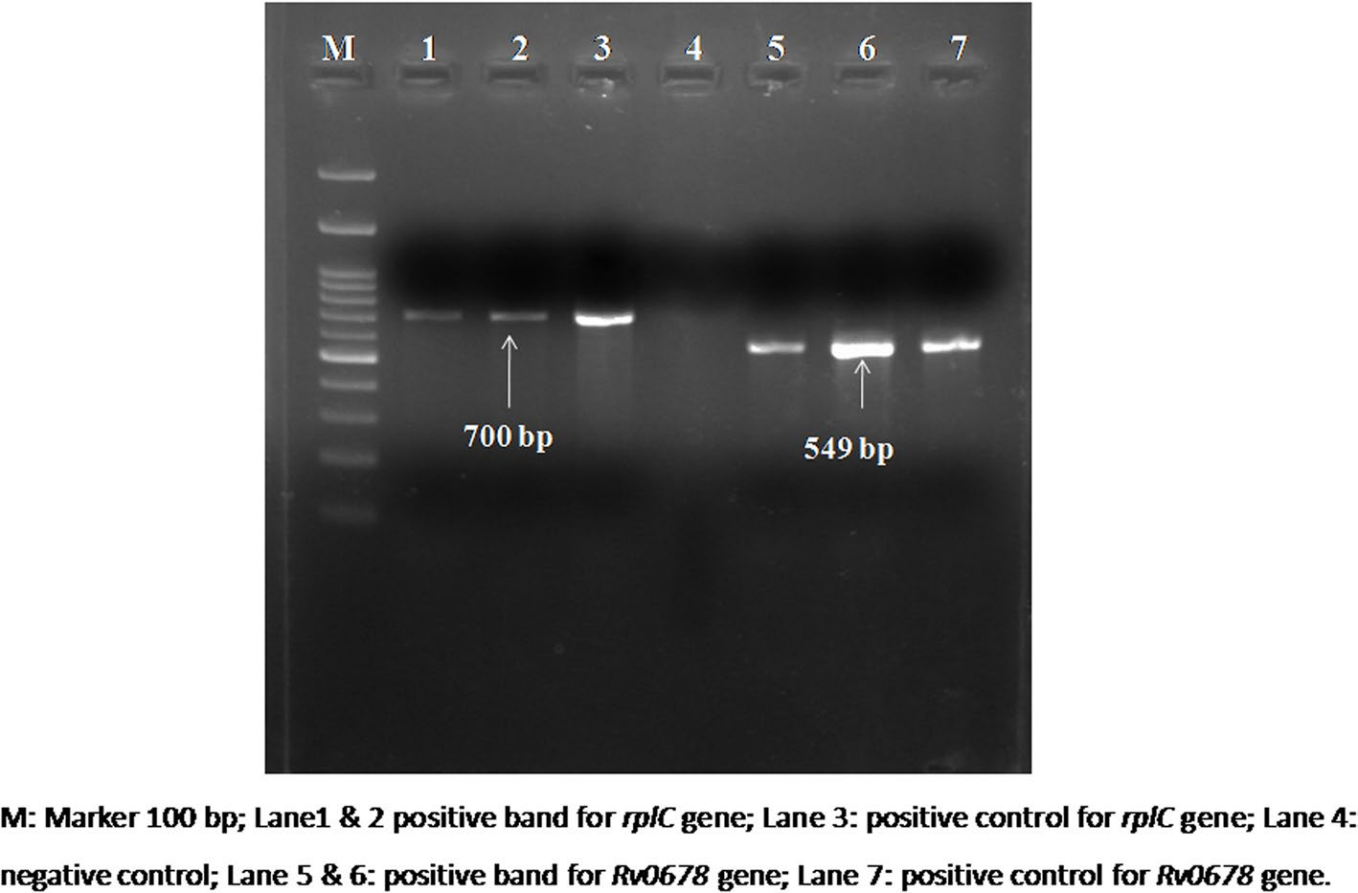
Gel-image showing amplified products of *rplC* and *Rv0678 gene*

Two isolates (K and J) showed resistance towards linezolid and clofazimine. The amplified product of *rplC* and *Rv0678* gene (Fig. 1) were sent for sequencing. The *rplC gene* sequencing for detection of linezolid resistance showed that there was a point mutation at position 460 bp, where thymine (T) is substituted for cytosine (C). This resulted in amino acid variation; cysteine in place of arginine at position 154. In the case of the *Rv0678* gene responsible for clofazimine resistance in both the isolates, there were seven mutations at the position from 46 to 220 bp. Substitution at each nucleotide leading to change in amino acids at positions, namely, isoleucine 16 leucine, glutamic acid 18 aspartic acid, phenylalanine 27 leucine, leucine 56 arginine, alanine 61 proline, threonine 69 proline, leucine 74 valine (I16L, E18D, F27L, L56R, A61P, T69P, and L74V). However, the most crucial change seen in the *Rv0678* gene was the deletion of guanine (G) nucleotide at 307 bp and 308 bp, resulting in frameshift from 103 amino acid leading to stop codon after 104^th^ amino acid in both isolates. Besides these mutations, there were 14 more mutations noted within the *Rv0678* gene between nucleotide positions 326 bp to 496 bp. Nine mutations were present in both isolates (K and J), and five mutations were only in the J isolate. Although, these mutations did not affect because frameshift had appeared earlier to these mutations resulting in truncated protein after 104^th^ amino acid.

### Phenotypic confirmation for resistance

In phenotypic confirmation of resistance, only two isolates showed visible growth on Lowenstein Jensen (LJ) media and liquid culture for tubercular bacilli.

## Discussion

Drug resistance has become a significant problem in the management of TB, with an urgent need for research into new drugs. In this study, we got broadly similar results of linezolid and clofazimine DST for MTBC obtained by 7H10 agar dilution and REMA. The MIC_50_ and MIC_90_ obtained for linezolid were 0.5 µg and 1 µg whereas, for clofazimine, the MIC_50_ and MIC_90_ were 1 µg for both. The essential and categorical agreement for linezolid was 97.63% and 95.26% and for clofazimine both were 100%. Further, the much shorter turnaround time for REMA (approximately seven days) is a significant advantage over the agar dilution method. Similarly, a study from the Netherlands showed that the 7H10 agar dilution and MGIT 960 phenotypic second-line DST methods for *M. tuberculosis* yielded essentially identical results, except for prothionamide. For moxifloxacin and clofazimine, they proposed 0.5 µg/ml and 1 µg/ml, respectively, as breakpoint concentrations for the MGIT 960 method. They also determined the MIC_90_ for moxifloxacin (0.5 µg (7H10) 0.25 µg (MGIT) and clofazimine (0.25 µg) [11]. According to T. Schön et al*.,* 2011 the tentative epidemiological wild-type cut-offs (ECOFF) were determined by using a 96-stick replicator in Middlebrook 7H10 medium for clofazimine and linezolid in consecutive susceptible clinical isolates (*n* = 78). They found that the wild-type MIC distribution was 0.64 to 0.125 mg/l (ECOFF = 0.125 mg/l). Only one isolate was above the ECOFF; this strain was resistant to amikacin, kanamycin, and capreomycin, but was susceptible to all first-line drugs. While for linezolid the wild-type MIC distribution ranged from 0.125 to 0.5 mg/ml (ECOFF = 0.5 mg/l) [18]. In a study from Pakistan, a total of 102 MTB isolates (XDR, *n* = 59; pre-XDR, *n* = 43) were used to determine susceptibilities by the Middlebrook 7H10 agar method. Based on the MIC cut-off (0.5 µg/ml) used for linezolid in the present study, 5.9% (6/102) of the strains tested were found to be resistant (i.e., linezolid MIC ≥ 1.0 µg/ml). These linezolid-resistant isolates belonged to both XDR and pre-XDR groups (3 from each group). For 94.1% of the MTB isolates, the linezolid MIC was ≤ 0.5 µg/ml, and therefore these strains were considered susceptible to linezolid. Only for one XDR isolate the linezolid MIC found to be 2 µg/ml [8]. Weiss et al*.,* 2015 conducted the in vitro susceptibility tests of linezolid by determination of MICs against 148 MTB strains including 18 MDR-TB strains isolated from 2002 to 2012. The testing for MIC was performed on solid Middlebrook-7H10 agar plates. They found MICs for 18 MDR-TB-strains in the range of 0.125–0.5 μg·mL − 1 and 130 non-MDR-TB strains between 0.125–0.5 μg·mL − 1 [19]. According to Kaniga et al., 2016, a multi-laboratory study was conducted to determine MIC quality control (QC) ranges for Phenotypic Drug Susceptibility Testing of linezolid (0.25 to 2 g/ml) and clofazimine (0.03 to 0.25 g/ml) [20]. In another study from China, the MIC was determined by the alamar Blue assay in 90 XDR-TB strains. They found the breakpoint MIC for resistance (mg/liter) against bedaquiline, delamanid, linezolid, clofazimine, moxifloxacin, and gatifloxacin were as follows: 0.25, 0.125, 1, 1, 0.5, and 0.5 respectively [21]. According to Nimmo et al*.,* 2020 clofazimine MICs for isolates with wild-type *Rv0678* genes ranged from 0·12 to 0·5 µg/mL, while those with *Rv0678* variants ranged from 0·25 to 4·0 µg/mL [22].

The present study also showed that in two isolates (K and J), the sequencing result of the *rplC* gene revealed a point mutation at position 460 bp where T is substituted for C. This resulted in amino acid variation; cysteine for arginine at position 154. In the case of the *Rv0678* gene in both isolates there were, 7 mutations at 46 bp to 220 bp. Substitution at each nucleotide led to change in amino acids at positions, namely, I16L, E18D, F27L, L56R, A61P, T69P, and L74V. Pang et al*.,* 2017 reported the mutation in the *Rv0678* gene in four clofazimine resistant strains where amino acid substitution at 53 codon (Ser53Pro) and 157 codon (Tyr157Asp) were observed. Further investigating the mutations associated with linezolid resistance, they targeted *23S rRNA*, *rplC*, and *rplD* among 5 linezolid resistant strains. Of the 5 strains, only 2 had mutation where amino acid substitution occurred at position 154(Cys154Arg) in the *rplC* gene, while the other two genes seemed not to account for linezolid resistance [21]. According to Beckert et al*.,* 2012, the T460C mutation in the *rplC* gene was the most frequent among the linezolid resistant isolates [23]. Recently a study from China in 2021 found a similar result [24].

## Conclusion

Therefore, our study clearly suggests that the lesser turnaround time for REMA has an advantage over agar dilution method. Importantly, the mutations in *rplC* and *Rv0678* gene were responsible for drug resistance to linezolid and clofazimine respectively.

## Materials and methods

This prospective study is the extension of our previous work [25], conducted over a period of 1 year (January 2019 to December 2019). The study is a part of routine diagnostic workflow under NTEP (National TB Elimination Program) where all presumptive TB sputum specimens were collected and transferred to Culture and DST laboratory, Department of Microbiology, Institute of Medical Sciences, Banaras Hindu University, Varanasi, Uttar Pradesh. A total of 188 XDR-TB sputum specimens were confirmed with the help of following genotypic methods:

- **GeneXpert assay (Cepheid)** is an automated, cartridge-based real-time PCR system, which is used for the detection of MTBC along with rifampicin (RIF) resistance. GeneXpert MTB/RIF assay detects rifampicin resistance by targeting the *rpoB* gene [25].
- **GenoType MTBDR plus assay (Hain Liefescience, Nehren Germany)** identifies MTBC and detects resistance to rifampicin (RIF) and isoniazid (INH). It detects MTBC directly from sputum or liquid or solid culture and mutations in the *rpoB* gene conferring RIF resistance, *katG* gene conferring high-level INH resistance, and the *inhA* gene conferring low-level INH resistance, through PCR and reverse hybridization [25].
- **GenoTypeMTBDR** ***sl*** **assay (Hain Liefescience, Nehren Germany)** which detects MTBC and multiple mutations which are associated with resistance to fluoroquinolones (FQs) and second-line injectable drugs (SLIDs). Mutations in *gyrA* and *gyrB* are detected for resistance to FQs, while resistance to SLIDs are detected through mutations in *rrs* and *eis* gene were included [25].

### Specimen processing

The collected sputum specimens were decontaminated as described elsewhere [26, 27]. Briefly, specimens were decontaminated using N-acetyl-L-cysteine and sodium hydroxide (NALC-NaOH) method. The decontaminated and concentrated sediments (0.5 ml) were inoculated into the BACTEC MGIT 960 (BD, USA) automated liquid culture system, used for early detection of mycobacterial growth, and drug sensitivity testing. Further 0.2 ml was inoculated into Lowenstein Jensen (LJ) media, which is an egg based selective solid medium used for the isolation of MTBC [26]. The cultures which gave a positive result on BACTEC MGIT 960/LJ, Ziehl–Neelsen (ZN) smear and capilia test (An MPT64 based, rapid immuno-chromatographic identification method, used for the confirmation of MTBC from MGIT 960 instrument positive) with no growth on Brain Heart Infusion agar (BHI) were included in this study [27].

### Preparation of antimicrobial stock solution

Linezolid and clofazimine drug powder were purchased from Sigma-Aldrich chemical, Ltd, India. The stock solutions were prepared according to the drug potency using the following formula:

$$\mathrm{Weight}\;\left(\mathrm{mg}\right)\;=\frac{\mathrm{Volume}\;\mathrm{required}\;(\mathrm{mL})\;\times\;\mathrm{Desired}\;\mathrm{drug}\;\mathrm{concentration}\;(\mathrm{mg}/\mathrm{ml})\;\times\;1000}{\mathrm{Potency}\;(\mathrm{mg}/\mathrm g)}$$

The calculated amount of drug was dissolved in dimethyl sulfoxide (DMSO), an aliquot of the stock solution was used for each test [28].

### Inoculum preparation

A loopful (10 µl plastic inoculating loops, Tarsons Products Pvt. Ltd., India) of isolated colonies was suspended in 5 ml normal saline. To ensure biosafety, the inoculum was prepared in unbreakable plastic falcons. The suspensions were homogenized by shaking with glass beads for 20 min at 350 rpm in a homogenizer. To allow sedimentation, the suspensions were incubated at room temperature for 10 min. The turbidity of the supernatant was adjusted by adding sterile normal saline according to 1 McFarland standard to obtain a density of 2 × 10^5^ to 10 × 10^5^ CFU/ml [28].

### Agar dilution method

Middlebrook 7H10 media (HiMedia Laboratories Pvt. Ltd. India) was used for MIC determination by the agar dilution method. The media was prepared as directed by the manufacturer and sterilized by autoclaving. The media was allowed to cool around 45 °C at room temperature. The petri plates were labeled properly with antimicrobial concentration to be poured. The antibiotic solution was added to the sterilized melted Middlebrook 7H10 medium, with the following twofold serial dilution concentrations: 0.125, 0.25, 0.5, 1, 2 µg/ml for linezolid (sigma) and for clofazimine (sigma) 0.25, 0.5, 1, 2, 4 µg/ml. The range of concentration was selected according to previous studies [11, 20, 29]. Approximately 25 ml media with particular antibiotic concentration was poured onto respective petri plates. The plates were allowed to set at room temperature so that no drops of moisture remained on the surface of the agar. The prepared inoculum was diluted 1:10 in sterile saline and inoculated onto the antibiotic plates with the help of sterile swab sticks. All the processing was done in a Class II biosafety cabinet in the BSL3 laboratory. The plates were allowed to stand at room temperature until the moisture in inoculum spots was absorbed into the agar. The plates were then kept for incubation at 37 °C. The MIC values were noted and interpreted according to WHO guidelines and previously described studies [11, 20, 29].

### Resazurin microtiter assay (REMA)

The REMA plate assay was carried out as described by Palomino et al. Briefly, 100 µl of 7H9 broth was dispensed in each well of a sterile 96-well plate, and serial twofold dilutions of each drug were prepared directly in the plate. The drug concentration ranges used were as follows: for linezolid 0.125, 0.25, 0.5, 1, 2 µg/ml and 0.25, 0.5, 1, 2, 4 µg/ml for clofazimine. One hundred microliters of prepared inoculum were added to each well. Growth control and a sterile control were also included for each isolate. Sterile water was added to all perimeter wells to avoid evaporation during the incubation. The plate was covered, sealed in a plastic bag, and incubated at 37 °C. After seven days of incubation, 30 µl of resazurin solution was added to each well, and the plate was re-incubated overnight. A change in color from blue to pink indicated the growth of bacteria. The MIC was defined as the lowest concentration of drug that prevented this color change [16].

### Quality control strain and growth control

*Mycobacterium tuberculosis* H37Rv susceptible to all standard anti-tuberculosis agents was taken as a control strain. The plates containing only media and inoculum were taken as growth control.

### Phenotypic confirmation for resistance

The isolate found resistant to a particular drug with visible growth on the antibiotic plate was confirmed by subculturing into the LJ media and liquid culture for the presence of tubercular bacilli.

### MIC_50_ and MIC_90_ values determination

The MIC_50_ and MIC_90_ values and the range of values obtained through MIC are essential parameters for reporting results of susceptibility testing when multiple isolates of a given species are tested. The MIC_50 _and MIC_90 _represent the MIC value at which ≥ 50% and ≥ 90% of the isolates in a test population were inhibited. MIC_50_ and MIC_90_ were defined for each mutant group against linezolid and clofazimine by using the following formula: MIC_50_ = no. of isolates (*n*) × 0.5 and MIC_90_ = no. of isolates (*n*) × 0.9 [30, 31].

### Statistical analysis

Essential agreement for linezolid and clofazimine was calculated as % of isolates producing MICs that are within ± 1 doubling dilution of the agar dilution method (standard method) and categorical agreement for both was calculated as % of isolates producing same category result compared to MIC by agar dilution method (standard method). The error rates were based at each MIC on the discrepancies in MIC by REMA method, as compared to agar dilution method [32].

### Mutation detection

**Fig. 2:**
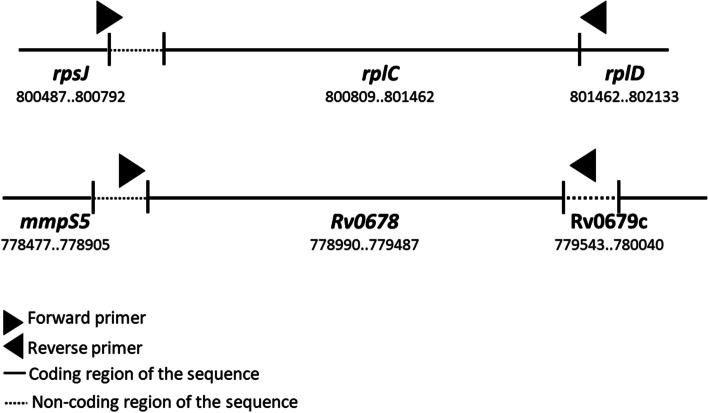
Schematic representation of primer position for gene amplification

The isolates which showed non-susceptibility towards linezolid and clofazimine were further subjected to PCR for mutation detection. Genomic DNA was extracted by the CTAB-chloroform method. The quality and quantity of DNA were analyzed with the help of a spectrophotometer (Thermo Scientific NanoDrop 2000). Primers were designed to amplify the *rplC* (Linezolid) and *Rv0678* (clofazimine) genes from flanking region by simplex PCR (Fig. 2, Table 4).The reaction mixture was prepared containing 2.5 µl of 10X reaction buffer (GeNei, Bangalore, India), 2 µl of 200 M concentrations of each of the deoxynucleoside triphosphates (dNTPs) (GeNei, Bangalore, India), 0.3 µl of 5 U Taq DNA Polymerase (GeNei, Bangalore, India), 1 µl of each oligonucleotide primers forward and reverse (10 pmol each) (GeNei, Bangalore, India), 5 µl (50 ng) of the DNA template and milli Q to maintain the final volume of 25 µl.

**Table 4:** Oligonucleotide used as a primer for amplification

S.No	Target Gene	Target Drug	Primer Sequences (5ˈ- 3ˈ)	Product Size(bp)	Reference
1	*rplC gene*	Linezolid	CAGTAGGAGATTGGACAGA TGTCTTCTGCTCTTGCGC	700	This study
2	*Rv0678 gene*	Clofazamine	CGTCACAGATTTCAGAGTACA GTCAGATTGCGAGGTTGCT	549	This study

### PCR running conditions

Initial denaturation step at 95 °C for 15 min followed by following parameters:

$$\left.\begin{array}{c}\mathrm{DNA}\;\mathrm{denaturation}\;\mathrm{at}\;95^\circ\mathrm C\;\mathrm{for}\;30\;\mathrm{second}\\\mathrm{Primer}\;\mathrm{annealing}\;\mathrm{at}\;62^\circ\mathrm C\;\mathrm{for}\;45\;\mathrm{second}\\\mathrm{Extension}\;\mathrm{at}\;72^\circ\mathrm C\;\mathrm{for}\;45\;\mathrm{seconds}\end{array}\right\}30\;\mathrm{cycles}\\\mathrm{Final}\;\mathrm{extension}\;\mathrm{step}\;\mathrm{at}\;72\;^\circ\mathrm C\;\mathrm{for}\;5\;\mathrm{minutes}$$

### Sequencing of *rplC* and *Rv0678* gene

The *rplC* and *Rv0678* gene was amplified with the help of primers, as shown in Table 4. Product size was confirmed by agarose gel electrophoresis. The amplified PCR products were purified using QIAquick® PCR and Gel Cleanup Kit (Qiagen India Pvt. Ltd, India) and sent for Sanger sequencing to Eurofins Genomics Pvt Ltd, India. The sequencing data has been deposited in the GenBank repository (accession numbers ON160017, ON160018, ON160019, and ON160020).

### Sequence data analysis

The sequence of resistant isolates to both linezolid and clofazimine along with one control (H37Rv) for each were analyzed by using BioEdit version 7.0.5.3 software tool [27, 33]. All the mutant sequences were compared with the control (H37Rv) sequence by using Clustal W multiple sequence alignment on BioEdit software, and the mutations in the nucleotide sequences were marked. After nucleotide sequence analysis, both the control (H37rv) and mutant nucleotide sequences were in-vitro translated on ExPASY translate (https://web.expasy.org/translate/). The in-vitro translated sequences of both control (H37Rv) and mutant proteins were also analyzed by Clustal W multiple sequence alignment on BioEdit software. The mutations in the protein sequences were marked.

## Supplementary Information

**Additional file 1.**

**Additional file 2.**

## Data Availability

All data generated or analysed during this study are included in this published article (and its supplementary information files) and are available in the GenBank repository, accession numbers ON160017**,** ON160018, ON160019, and ON160020.

## Abbreviations

MDR-TB: Multidrug-resistant tuberculosis
XDR-TB: Extensively drug-resistant tuberculosis
MIC: Minimum inhibitory concentration
REMA: Resazurin microtiter assay
DR-TB: Drug-resistant tuberculosis
DST: Drug susceptibility testing
MTBC: *Mycobacterium tuberculosis complex*
CC: Critical concentration
pWT: Phenotypically wild type
pNWT: Phenotypically non-wild type
ECOFF: Epidemiological wild-type cut-offs
QC: Quality control
NALC-NaOH: N-acetyl-L-cysteine and sodium hydroxide
ZN: Ziehl–Neelsen
BHI: Brain heart infusion agar
dNTPs: Deoxynucleoside triphosphates
