# Supplementary figures and images for: Mutation detection and minimum inhibitory concentration determination against linezolid and clofazimine in confirmed XDR-TB clinical isolates
Source: BMC Microbiol. 2022 Oct 3;22:236. doi: 10.1186/s12866-022-02622-x (PMC9531458; doi:10.1186/s12866-022-02622-x)

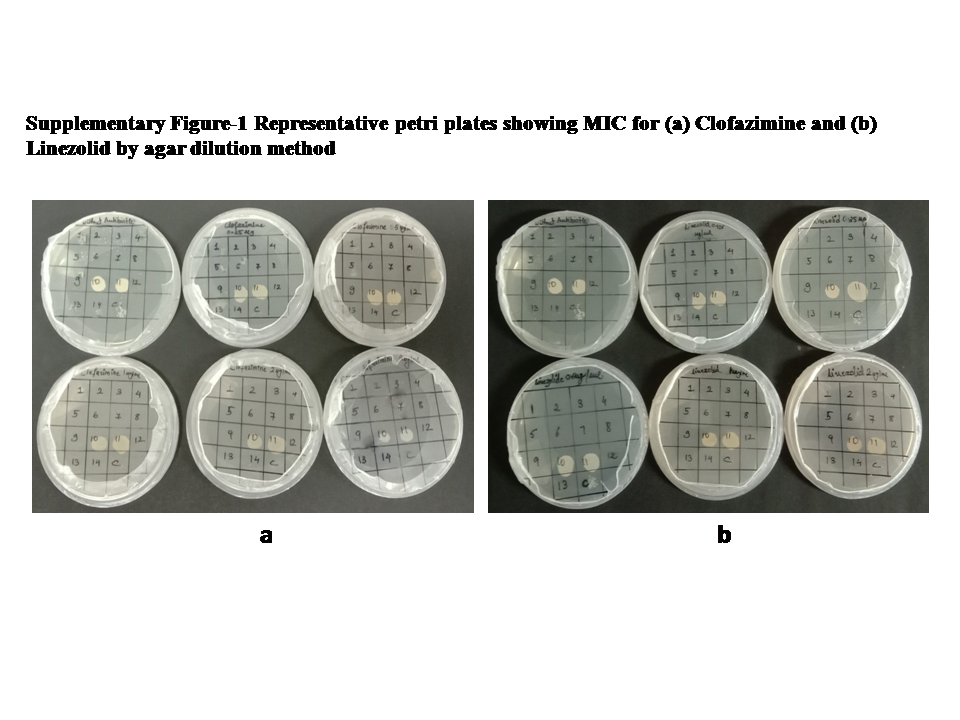

Supplement: Supplementary file 1 — Additional file 1. [file 12866_2022_2622_MOESM1_ESM.tif]

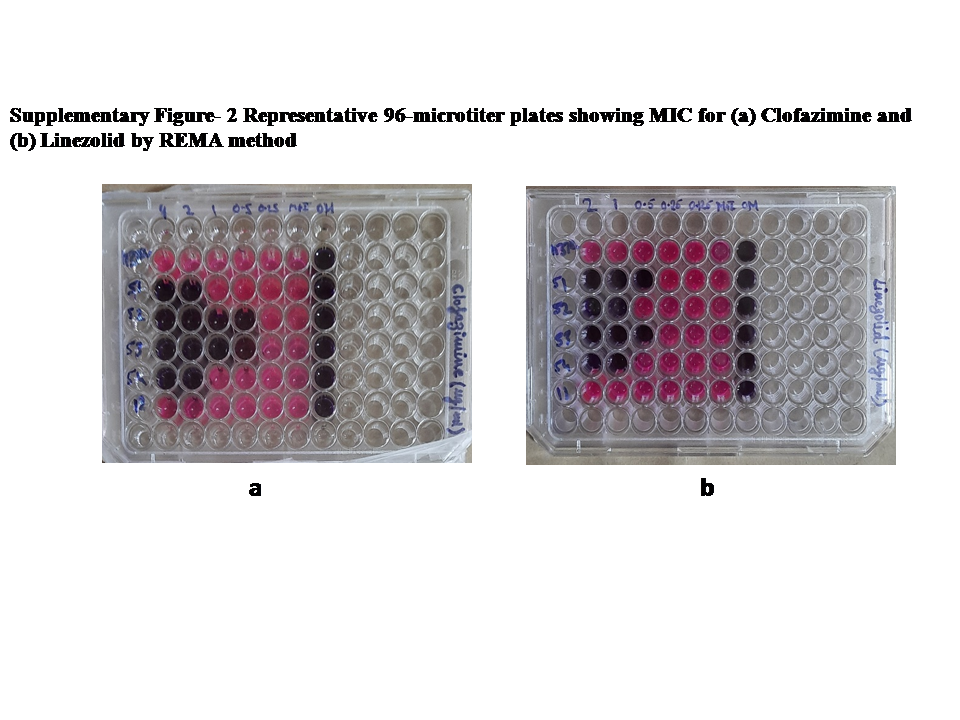

Supplement: Supplementary file 2 — Additional file 2. [file 12866_2022_2622_MOESM2_ESM.tif]
